# Vigor Encoding in the Ventral Pallidum

**DOI:** 10.1523/ENEURO.0064-21.2021

**Published:** 2021-08-18

**Authors:** James Lederman, Sylvie Lardeux, Saleem M. Nicola

**Affiliations:** Departments of Neuroscience and Psychiatry, Albert Einstein College of Medicine, Bronx, NY 10528

**Keywords:** accumbens, *in vivo* electrophysiology, motivation, reward, ventral pallidum

## Abstract

The ventral pallidum (VP) is the major downstream nucleus of the nucleus accumbens (NAc). Both VP and NAc neurons are responsive to reward-predictive stimuli and are critical drivers of reward-seeking behavior. The cue-evoked excitations and inhibitions of NAc neurons predict the vigor (latency and speed) of the cue-elicited locomotor approach response and encode the animal’s proximity to the movement target, but do not encode more specific movement features such as turn direction. VP neurons also encode certain vigor parameters, but it remains unknown whether they also encode more specific movement features, and whether such encoding could account for vigor encoding. To address these questions, we recorded the firing of neurons in the VP of freely moving male rats performing a discriminative stimulus (DS) task. Similar to NAc neurons, VP neurons’ cue-evoked excitations were correlated with the speed of the upcoming approach movement and the animal’s proximity to the movement target at cue onset. Unlike NAc neurons, VP neurons’ firing reflected the efficiency of the approach movement path but not the latency to initiate locomotion. VP cue-evoked excitations are unlikely to be directly influenced by NAc cue-evoked excitations because unilateral treatment of the NAc with a dopamine D1 receptor antagonist, a manipulation that reduces NAc neurons’ cue-evoked excitations, did not alter ipsilateral VP cue-evoked excitations. These observations suggest that the two structures receive simultaneous activation by inputs conveying similar but not identical information, and work in parallel to set the vigor of the behavioral response.

## Significance Statement

The ventral pallidum (VP) connects the nucleus accumbens (NAc) and other upstream structures with downstream motor structures, providing a conduit by which motivation is translated to action. Prior studies have shown that the firing responses of VP neurons to cues that elicit motivated behavior reflect parameters related to the vigor of the motor response, but have not fully analyzed how firing could be related to a broader range of movement parameters. Here, we show that the speed and efficiency of locomotor approach to reward, as well as proximity to the reward-associated movement target, influence the firing of VP neurons independent of representation of other motor parameters. These results suggest a specific role for VP neurons in invigorating locomotor approach to reward-associated locations.

## Introduction

The ventral pallidum (VP) and the nucleus accumbens (NAc) are components of the basal ganglia that both play critical roles in the processing and execution of motivated behaviors. Anatomically, the VP is the major downstream target of medium spiny neurons (MSNs) in the NAc (Root et al., 2015). At least half of both D1 dopamine receptor-expressing and D2 receptor-expressing MSNs project to VP (Kupchik et al., 2015). Other inputs to VP arise from medial dorsal thalamus, medial prefrontal cortex, dorsal raphe, the ventral tegmental area, and basolateral amygdala. Outputs from VP connect to many motor output-related structures including the thalamus. Thus, the VP has canonically been considered as a relay linking cognitive and emotional processes to motor output (Root et al., 2015).

Consistent with this idea, VP neurons contribute to motivated behaviors such as cocaine seeking (Root et al., 2010, 2012, 2015) and cued approach to natural rewards (Richard et al., 2016, 2018). Subsets of VP neurons are active during approach behavior (Root et al., 2013; Richard et al., 2016, 2018), with many showing a prominent cue-evoked excitation that is greater in response to cues that predict reward than to those that do not, and greater in trials that elicit locomotor approach than in those that do not (Richard et al., 2016). Yet, the behavioral role of these excitations remains unclear. Some excitations exhibit correlations with vigor parameters such as the speed of movement and the latency to reach the movement destination (Richard et al., 2016, 2018). Although these results suggest that VP neurons’ cue-evoked excitations influence movement vigor, it is unknown whether the excitations reliably precede movement, as would be expected for firing that influences movement vigor. In addition, it is not known whether other features of movement (such as turn direction) or aspects of the behavioral situation (such as time since last reward) influence cue-evoked excitations. Such effects would imply that the behavioral role of VP cue-evoked firing responses is different from simply invigorating the subsequent approach behavior. Finally, it remains unknown whether the vigor-related firing of VP neurons is because of an underlying correlation with other movement parameters, which themselves are related to vigor.

To answer these questions, we recorded the firing activity of VP neurons as rats performed a discriminative stimulus (DS) task, and used video tracking of head-mounted LEDs to measure movement parameters. We then used multiple regression analysis to determine which of several parameters related to cue-elicited approach movement is reflected in the firing of VP neurons. We show that cue-evoked excitations in the VP are correlated with vigor parameters (speed and efficiency of movement) as well as proximity to the movement target but not other movement parameters.

These regression results are similar (but not identical) to those observed previously for cue-evoked excitations in the NAc (McGinty et al., 2013; Morrison et al., 2017). To investigate whether NAc cue-evoked excitations influence those in the VP, we recorded from VP neurons in rats performing the DS task and microinjected the dopamine D1 receptor antagonist SCH-23390 into the NAc, a manipulation that reduces cue-evoked excitations in the NAc (du Hoffmann and Nicola, 2014). We show that unilateral SCH-23390 infusions have little if any effect on DS task performance or VP cue-evoked excitations, whereas bilateral infusions reduce both behavioral responding to DS presentations and the firing responses of VP neurons to these cues. Together, these results suggest that the VP and NAc represent different but overlapping sets of vigor parameters and control cued approach vigor in parallel.

## Materials and Methods

All procedures were performed in accordance with the National Institutes of Health *Guide for the Care and Use of Laboratory Animals* and were approved by the Institutional Animal Care and Use Committee.

### Behavior

**Figure 1.**
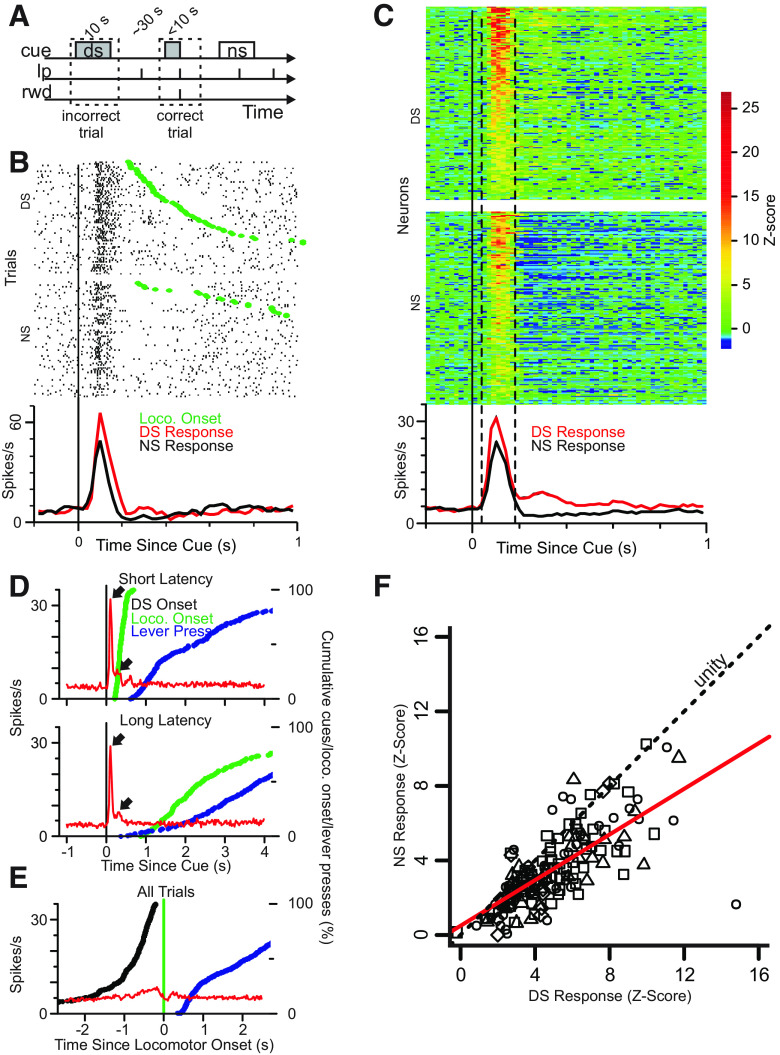
Ventral Pallidal neurons are cue-responsive and their firing reflects cue value. ***A***, Illustration of the behavioral task. Lp, lever press; rwd, reward; cue, DS or NS. ***B***, Raster plot (upper panels) showing firing aligned to DS and NS onset for a representative neuron. Movement onset is depicted for each trial by a green dot. Histogram (lower panel) shows mean firing rate across all correct trials for DS (red) and NS (black). ***C***, Heatmaps (upper panels) depict DS and NS response for all cue-excited neurons (*N* = 165). Color represents Z-score of firing rate as compared with a 1-s precue baseline. The lower panel shows the mean frequency of firing rate for neurons significantly excited by the DS. ***D***, Mean firing response to DS aligned to DS onset for first-quartile (shortest) movement onset latency (upper plot) and fourth-quartile (longest) movement onset latency trials (lower plot). Red line depicts average cue-evoked firing on DS trials in 98 DS-excited neurons. Blue and green lines depict cumulative percentage of locomotor onsets and lever presses, respectively. Note that the firing response to DS onset is similar in the two latency conditions, indicating that little to none of the excitation is because of a secondary excitation at movement onset. ***E***, Same as ***C*** but firing is aligned to movement onset for all trials. ***F***, Correlation of normalized (Z-score) response to the NS versus DS (40- to 180 ms window following cue-onset) for each neuron. Shapes indicate the animal from which the neuron was recorded. Red line indicates best-fit linear regression (*r*^2^ = 0.52). Slope <1 indicates that neurons respond more strongly to DS than NS.

After food restriction to 90% of normal body weight, freely moving male Long–Evans rats (300 g at arrival) were trained to associate a DS with availability of a liquid sucrose reward contingent on pressing a designated operant lever before cue-offset (<10 s; Ambroggi et al., 2008; Nicola, 2010; du Hoffmann and Nicola, 2014). Upon a lever press during the DS, the DS was terminated and reward was delivered in a nearby receptacle. A second, “inactive” lever was also present throughout the experiment. A separate fixed length 10-s cue (neutral stimulus; NS) was presented randomly interleaved with DS presentations. The intertrial interval (ITI) was exponentially distributed to approximate a constant probability of cue onset at all times. The mean ITI was 30 s (Fig. 1*A*). Active lever responses during the NS and ITI, and inactive lever responses at all times, were detected but had no programmed consequence. Cues consisted of siren (4- to 8-kHz ramping, 200-ms cycle time) and intermittent (6 kHz, 80-ms pulse duration, 200-ms cycle time) auditory stimuli. Each rat was randomly assigned one of these stimuli as the DS and the other as the NS; the assignment remained the same across training and experiments.

### Neural and video data recording

A total four rats that were trained and implanted with microelectrode arrays contributed neural data in these experiments. After rats were trained to a criterion level of performance (response ratio of 80% or greater), drivable microelectrode arrays were surgically implanted into the VP (bregma A/P: 0.12 mm, D/V: 8.20 mm, M/L: ±2.40 mm), and infusion cannula were implanted into the NAc (relative to bregma A/P: 1.50 mm, D/V: 6.00 mm, M/L: ±1.60 mm). Following recovery, extracellular activity of single VP neurons was recorded from the arrays during task performance. Each neuron was recorded in only one session. For each subsequent session, the arrays were advanced by 30 μm to record from a new set of neurons. Two different-colored LEDs were mounted to the headstage to track each rat’s head position and orientation using an overhead camera and computerized tracking system (Plexon Cineplex, 30 frames/s, ∼1.5-mm spatial resolution) during task performance. Behavioral sessions were 3 h in duration. The mean ± SD number of DS presentations in each session was 131.2 ± 8.3, and the mean number of NS presentations was 133.9 ± 6.4. Trials in which the animal was moving at cue onset were discarded resulting in a mean of 82.8 ± 18.6 DS trials and 84.6 ± 17.2 NS trials used for analysis.

In a subset of experiments, either 2 μg of SCH-23390 or vehicle control solution was infused over 2 min either unilaterally or bilaterally into the NAc after a 45-min control period.

### Measurement of locomotor onset and offset and other behavioral and locomotor features

The frame-by-frame *x*- and *y*-positions of the red and green LEDs were extracted from the video data using CinePlex software (Plexon). Correction for lens distortion was computed using images of calibration grids in the behavioral arenas and applied to the tracking data using a custom script written in R. Frame-by-frame behavioral event data were merged with tracking data and used to compute all measurements of behavioral and locomotor parameters (Table 1) using custom scripts in R.

**Table 1:** Locomotor and behavioral variables

	Variable	Units
	Locomotor Variables	
1	Radial velocity with respect to operant lever (mean)	mm/s
2	Radial velocity with respect to operant lever (maximum)	mm/s
3	Radial velocity with respect to operant lever (SD)	mm/s
4	Speed (mean)	mm/s
5	Speed (SD)	mm/s
6	Speed (maximum)	mm/s
7	Move duration	s
8	Path length	mm
9	Latency to time of maximum speed	s
10	Latency to time of maximum acceleration	s
11	Maximum distance between actual path and best (straight line) path	mm
12	Turn efficiency (cumulative summed change in heading/net change in heading)	None
13	Angular velocity with respect to operant lever (SD)	Radians/s
14	Angular velocity with respect to operant lever (maximum)	Radians/s
15	Angular velocity with respect to operant lever (mean)	Radians/s
16	Signed change in head orientation	Radians
17	Radial velocity with respect to operant lever (mean)	mm/s
18	Path efficiency	None
	Variables measured at moment of cue onset	
19	The distance from the lever at movement-onset	mm
20	Time elapsed since last reward received	s
21	Time elapsed since last lever press	s
22	Time elapsed since last cue (DS or NS)	s
23	Head orientation with respect to lever	Radians

A total of 23 locomotor and behavioral variables were used for GLM analysis.

Because the interval between cue presentations was long and unpredictable, animals moved about the chamber during this interval such that they were in different starting locations at each cue presentation. Therefore, animals likely used a taxic (or “flexible”) approach strategy to reach the lever from the multiple starting locations (Nicola, 2010, 2016). To characterize these movements, we used video tracking of two head-mounted LEDs, which provided frame-by-frame information about head position and orientation. As described previously (McGinty et al., 2013), these data were used to calculate, for every frame *t*, a “locomotor index” (LI), the SD of change in position across a sliding 11-frame window:

(1) $$LI_{t}=SD(d_{t-4},\ldots ,d_{t},\ldots ,d_{t+4}),$$

where SD is the SD function, and *d*_A_ is the difference in Euclidean distance from the prior frame.

Locomotor onsets and offsets were detected by determining when the LI crossed threshold values determined by fitting a 3-Gaussian mixture to the distribution of locomotor index values over the course of each session. The locomotor onset threshold was defined as the intersection of the second and third Gaussian, and the locomotor offset was defined as the intersection of the first and second Gaussian. A stillness-before-onset criterion was used which required 70% of the locomotor index values in the 1 s (30 frames) before putative movement onset to be below the locomotor offset threshold. Locomotor onset was thus defined as the first frame of four consecutive frames in which the locomotor index exceeded the onset threshold after a period of stillness. To define cue-evoked approach movements, the point at which locomotor onset occurred after cue onset was determined as the movement start time; trials in which the animal was already moving at the time of cue presentation were not analyzed. Movement end time was defined by the frame in which either a lever press or reward port entry was recorded in the case of correct trials, or the first frame in which the locomotor index dropped below the offset criterion in the case of incorrect trials. This method of movement detection is similar to methods published in previous works (Golani et al., 2005; McGinty et al., 2013; Morrison et al., 2017).

Locomotor variables such as average speed, turn direction and velocity, path length, and others were calculated using the locomotor data between the time of locomotor onset after cue presentation and locomotor offset. Behavioral state variables (e.g., distance from lever, orientation to lever, time since last reward) were calculated by using locomotor data at the time of cue onset (Table 1).

### Neural data analysis

Following data collection and preprocessing, all subsequent analysis was done using custom scripts in the R programming language. The dataset and code are available on reasonable request.

Neurons excited by the onset of DS presentation were identified as having three or more consecutive 20-ms bins within the interval of 40–180 ms after DS onset in which the firing rate exceeded a 99.9% confidence interval calculated based on the neuronal activity in the 1 s before DS onset. The first bin after cue onset that exceeded this interval was considered to be the onset of the excitatory response.

Before applying regression models, we first identified which of the locomotor variables best describe the cue-induced movement toward the operant lever by performing exploratory principal component analysis (PCA) and factor analysis (FA) on the redundant set of locomotor variables (Table 1). We restricted our analysis to DS trials in which a correct lever response was made, ensuring that the cue value and reward outcome were the same across trials. A total of 98 neurons from non-infused and vehicle-infused groups were used in the generalized linear model (GLM) analysis. We used neurons from only these groups, as opposed to all neurons from the initial 45-min control period, as there were insufficient trials in the control period to fit the GLM model for most neurons.

To detect correlations between behavioral and locomotor variables (regressors) and DS-evoked neuronal activity (response), we applied a GLM:

(2) $$\ln\left(Y\right)=\beta_{0}+\beta_{1}x_{1}+\beta_{2}x_{2}...+\varepsilon ,$$

where *x*_1_ … *x_n_* are independent variables (regressors; e.g., movement speed), *β*_0_ … *β_n_* are the regression coefficients obtained from the fit models, *ε* is the residual (error) term, and *Y* is the cue-evoked spike count (the response variable). Note that the natural log transformation (link function) is not a transformation applied to the response data, but rather refers to the predicted values of the fitted model (Venables and Ripley, 2002). Neuronal spike counts are best fit by a Poisson distribution, for which the logarithmic link is appropriate.

To assure that the regression models used did not produce spurious results because of excessive multicollinearity among the independent variables, we computed an index of multicollinearity for each variable, the squared multiple correlation (SMC). We eliminated variables for consideration in GLM analysis with a SMC value over 0.8, a conservative tolerance for concern over the impact of multicollinearity on regression model fitting.

To facilitate comparison between the regression estimates, they were scaled and converted to the estimated percentage difference in firing rate over the interdecile range (IDR Firing Difference) of each regressor, using the following formula:

(3) $$\text{IDR Firing Difference }=\left({\text{e}}^{\beta_{A}}*IDR_{A}-1\right)*100\%,$$

where *β_A_* is the regression estimate for the locomotor variable *A*, and *IDR_A_* is the IDR of that variable (the difference between 90th and 10th percentiles).

Where appropriate, *p* values were corrected using Holm’s modified Bonferroni correction. 

### Histology

Rats were perfused with formalin. Their brains were removed, sectioned, and Nissl stained, and the electrode and cannulae tracks were reconstructed. 

## Results

Single-unit recordings were obtained from freely moving rats that were performing a discriminative-stimulus (DS) task. Rats were presented with one of two distinct auditory cues at variable intervals with a mean intercue interval of 30 s. One auditory cue, deemed the DS, signaled the availability of reward (10% sucrose solution) at the reward port, contingent on a correct “active” lever press. Presses on a second, “inactive lever” were recorded but had no programmed consequences. The two types of levers were present throughout the behavioral session. The DS was on for up to 10 s and terminated on active lever press. A NS was also presented for 10 s at variable intervals (mean 30 s), and active or inactive lever presses during the NS and the ITI had no programmed consequences. Lever identities remained fixed for each rat throughout training and experiments. Trained rats responded to nearly all DS presentations and few NS presentations, and locomotor onset latency was generally longer for NS responses than for DS responses (Table 2).

**Table 2:** Summary statistics by experimental subject

Subject	Sessions	Response ratio (DS)	Response ratio (NS)	DS, locomotor latency (s)		NS, locomotor latency (s)		DS, mean locomotor speed (m/s)		NS, mean locomotor speed (m/s)	
				μ	σ	μ	σ	μ	σ	μ	σ
1	7	0.93	0.11	1.16	1.66	2.10	2.07	0.34	0.13	0.29	0.13
2	11	0.92	0.10	1.39	1.66	2.24	2.10	0.36	0.15	0.33	0.14
3	5	0.91	0.13	1.06	1.41	2.82	2.60	0.26	0.11	0.23	0.08
4	2	0.90	0.06	1.27	1.49	2.62	2.42	0.37	0.13	0.31	0.11
Grand mean	0.92	0.10	1.22	1.56	2.45	2.30	0.33	0.13	0.29	0.12	

Data from four subjects were used for analysis in this study. This table shows the number of sessions from each subject that contributed data for analysis in this study as well as the response ratios, locomotor onset latency, and mean speed of approach to the operandum for each subject across all sessions for both DS and NS responses (defined as pressing the active lever operandum within 10 s of cue presentation).

After training, rats were implanted with electrodes and microinfusion cannulae. In a subset of experiments, the dopamine D_1_-receptor antagonist SCH-23390 or vehicle was infused either unilaterally or bilaterally via cannulae targeted to the NAc 45 min after the start of the session.

### Cue-evoked firing in VP neurons reflects cue identity, predicts response likelihood and precedes locomotion onset

A total of 416 neurons were recorded from four animals across 28 behavioral sessions. Of these neurons, 165 were significantly excited by DS onset (for criteria used to determine significance of response, see Materials and Methods). Rasters and histograms aligned to cue onset are shown for an example DS-excited neuron in Figure 1*B*, and Figure 1*C* shows a heat map of normalized firing aligned to cue onset for all 165 DS-excited neurons (recorded during sessions in which no injection was made in the NAc, or saline was injected in the NAc, or during the 45-min preinjection baseline before SCH-23390 injection in the NAc). Typically, the excitation onset latency was between 20 and 40 ms (64/165), or 40 and 60 ms (69/165) with the remainder (32/165) having a latency >60 ms. Nearly all of these neurons also showed significant activation in response to NS onset; however, the DS-evoked response (mean ± SD, 21.0 ± 9.8 Hz) was significantly larger than the NS-evoked response (14.6 ± 7.9 Hz; paired Wilcoxon signed-rank test, *p *<* *0.0001; Fig. 1*F*). A small subset of neurons (15/416) was significantly activated by the NS but not by the DS, but these were not further analyzed.

**Figure 2.**
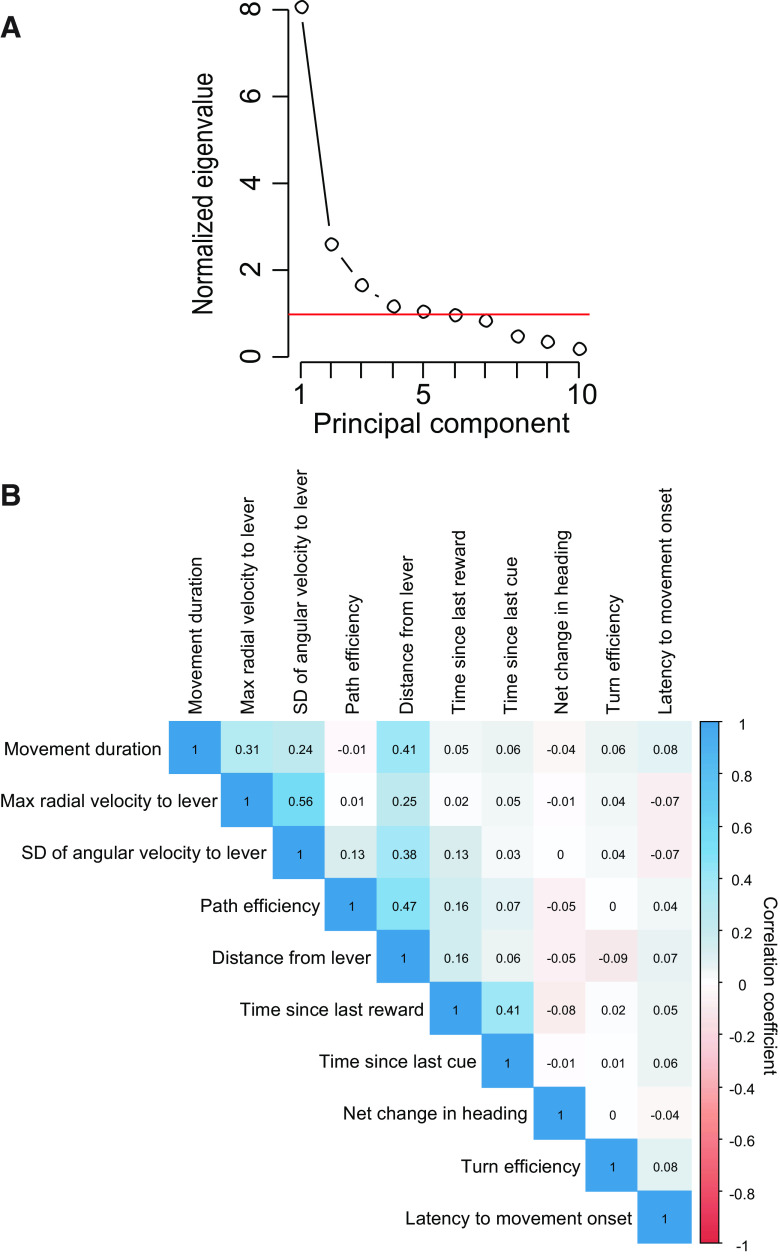
Scree plot of factor analysis on locomotor variables and cross-correlation coefficients of variables selected for GLM. ***A***, A scatter plot showing the normalized magnitude of the scalar component of each principal component (eigenvalue), where the mean value of all eigenvalues is 1. Because five components have values >1, we considered these five factors in subsequent FA of the data. ***B***, Correlation matrix showing the correlation coefficients for each pair of variables used in the GLM analysis.

**Figure 3.**
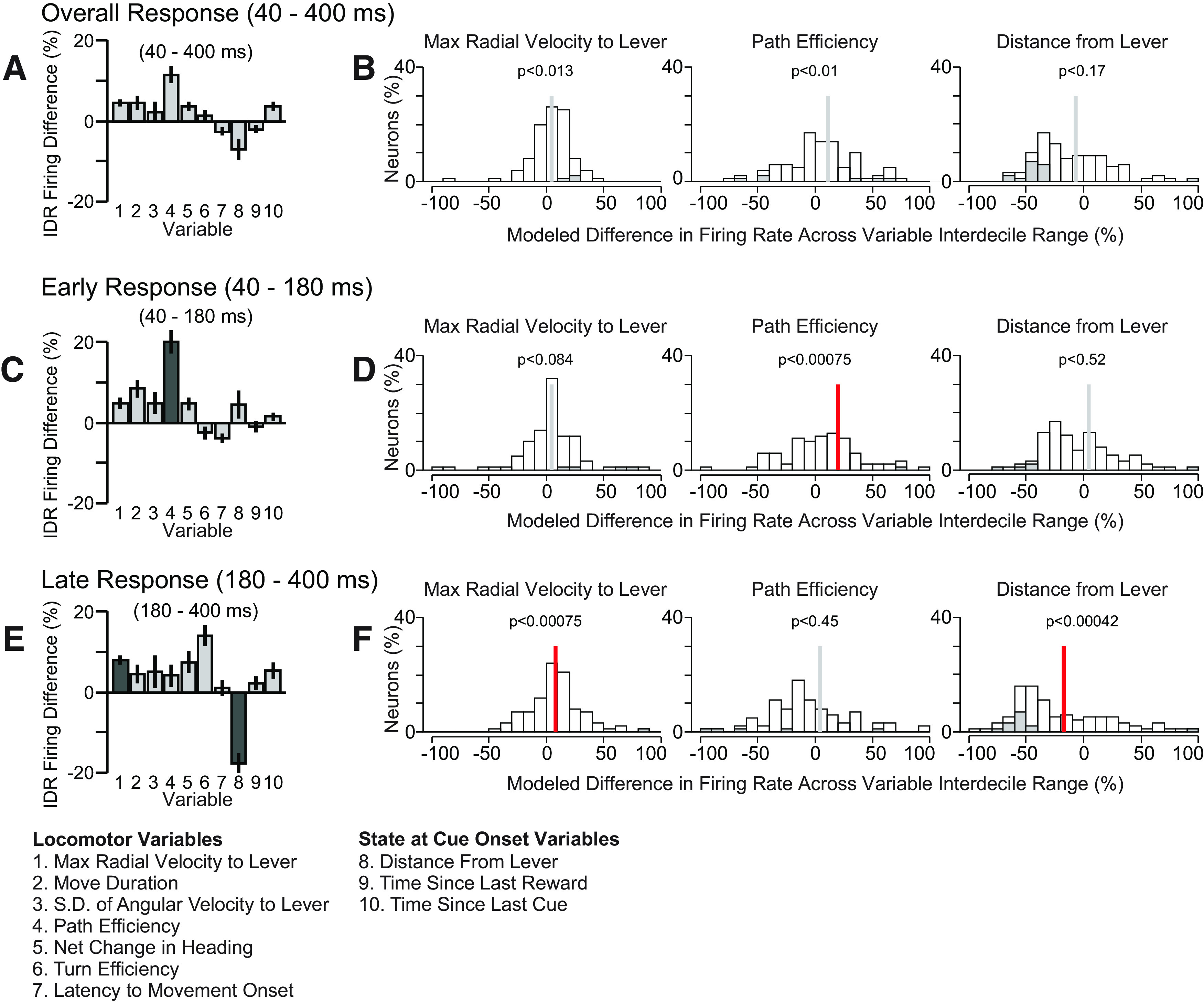
Cue-evoked firing of VP neurons was correlated with movement speed toward the lever and with lever proximity at cue onset. Analysis results from the larger (10-variable) GLM are shown. ***A***, Each bar indicates mean and SE of normalized regression estimates (IDR Firing Difference, the percent change in firing response across the IDR of the variable) for 165 cue-excited neurons in the overall firing response window (40–400 ms after DS onset). Dark shaded bars indicate significantly correlated regressors. ***B***, Individual distributions of three regressors across cue-excited neurons. Gray bars indicate individually significant coefficients. ***C***, ***E***, Same as ***A*** but for the early and late response windows, respectively. ***D***, ***F***, Same as ***B*** but for the early and late response windows, respectively. In ***B***, ***D***, ***F***, the vertical bar indicates the mean, and the color of the bar indicates whether the mean is significantly different from 0 (red, *p* < 0.05, one sample *t* test with Holm’s modified Bonferroni correction).

**Figure 4.**
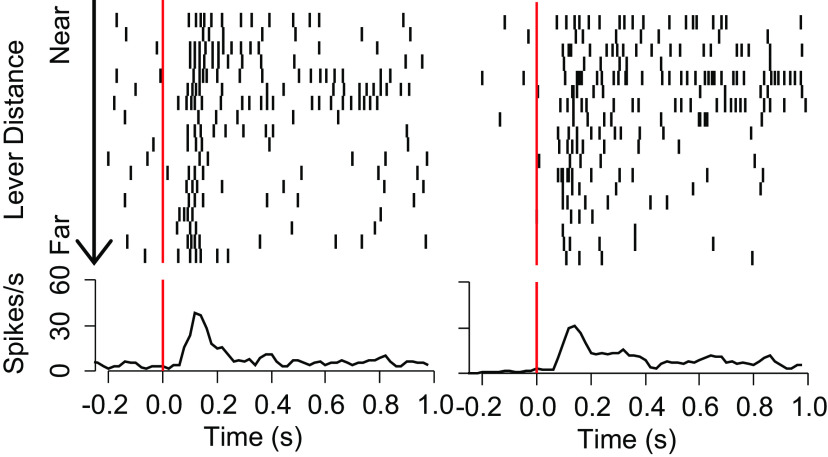
Representative neurons with firing related to distance from the lever at cue onset. Each raster/histogram pair shows one neuron’s firing in selected DS trials aligned to DS onset. Rasters show the time at which the neuron fires and are sorted from trials where the animal is closest to the lever (top) to farthest (bottom). Histograms show the mean firing rate across trials.

**Figure 5.**
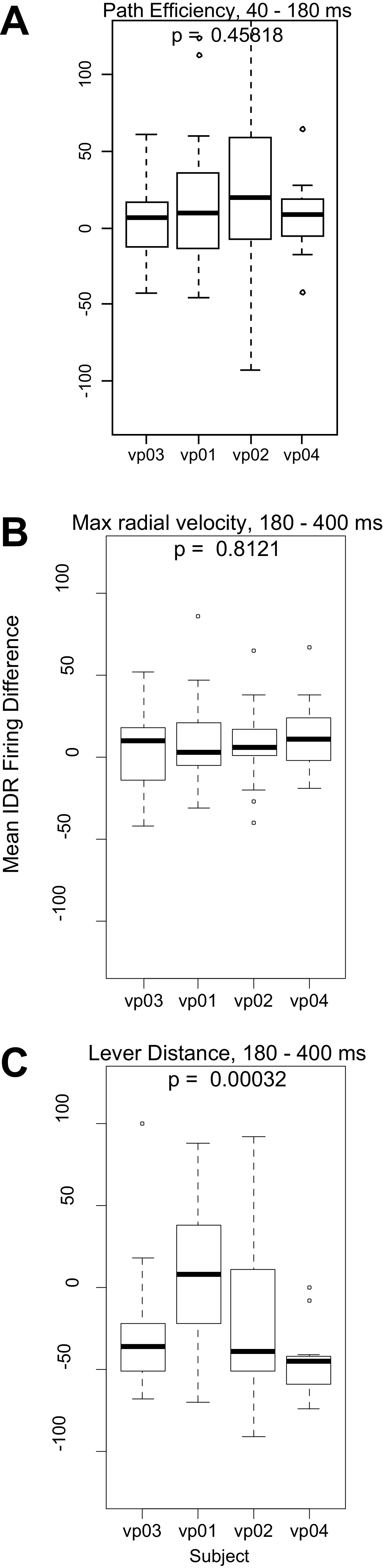
Comparison of IDR Firing Differences across rats. Each box-and-whisker plot describes an individual rat’s neurons’ IDR Firing Difference values for the indicated variable. The heavy horizontal line is the median, the box delimits the interquartile range, the lower whisker shows the smaller of the 25th percentile minus 1.5 times the interquartile range or the lower limit of the data range; the upper whisker shows the larger of the 75th percentile plus 1.5 times the interquartile range or the upper limit of the data range; and the individual points show values outside the whisker range; *p* values are the result of cross-subject ANOVAs.

**Figure 6.**
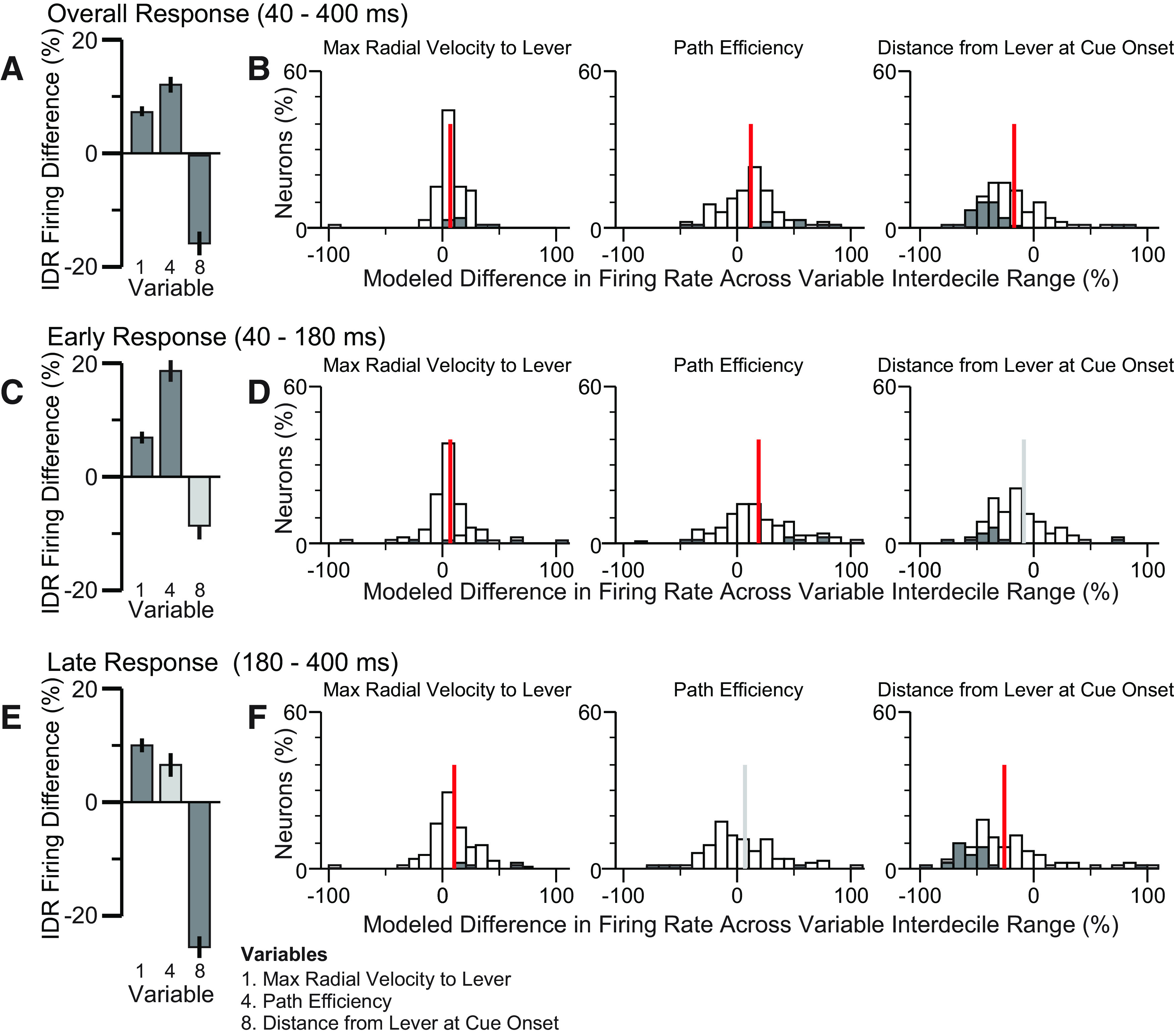
Focused GLM. Analysis results from the focused (three-variable) GLM are shown. Figure panels are as described for Figure 3.

We first determined whether post-DS firing was time-locked to cue onset or the onset of locomotion. We limited this analysis to trials in which the animal was still at cue onset so that we could identify the onset of locomotion after the start of cue presentation. Because this selection eliminated many trials, there was insufficient data from the 45-min preinjection baseline period to include neurons from sessions in which animals received drug injections. Therefore, we used the entire 3 h session but used data only from sessions in which animals received no injection or saline injection in the NAc. This yielded 212 total neurons, of which 126 were cue-excited; after eliminating two sessions in which the DS response ratio was <80% (44 neurons, 28 cue-excited), our final dataset consisted of 98 cue-excited neurons. Each rat contributed a similar number of neurons to this dataset (*N* = 26, 36, 23, and 13 neurons from the four rats). The analyses in Figure 1*D*,*E*, as well as the GLM analyses (Figs. 2–6) were performed on this 98-neuron dataset. To determine whether firing was aligned to DS onset or movement onset, we selected a subset of DS trials with active lever responses and >200-ms separation between cue onset and locomotion onset, and then further separated these trials into first and fourth quartiles of latency between cue onset and locomotion onset. When firing was aligned to DS onset, a short-latency, large-magnitude excitation was readily apparent, and the sharp alignment to cue onset (∼50-ms latency to peak firing) was similar across the range of locomotor latencies (Fig. 1*D*, upper arrows). There was an additional, smaller firing peak at ∼280 ms (Fig. 1*D*, lower arrows), the latency of which was also similar across locomotor latencies. This pattern of excitation was similar regardless of the locomotion onset latency (Fig. 1*D*, upper vs lower graphs, first and fourth latency quartiles, respectively). When these trials were aligned to locomotion onset, the magnitude of the excitatory response was much smaller than the cue-aligned firing (Fig. 1*E*), indicating that VP neurons’ cue-evoked excitations are strongly aligned to cue onset rather than initiation of movement.

To determine whether there was a substantial population of neurons that fired consistently at movement onset but not cue onset, we aligned the firing of all neurons that were not DS-excited to movement onset after DS presentation. There was no evidence of increased or decreased firing in this population aligned to movement onset (data not shown).

In neurons significantly activated by the DS, cue-evoked firing was greater on trials in which a correct operant response was made (8.4 ± 6.2 Hz) compared with when no response was made (5.9 ± 5.4 Hz; *p* < 0.001, paired Wilcoxon test; *n* = 163 neurons from 12 sessions in which there was at least 1 missed DS trial). Therefore, because cue-evoked activity consistently precedes locomotor initiation, and this firing is greater when the animal responds than when it does not, cue-evoked excitations in the VP could influence the initiation or maintenance of cued reward-seeking behavior.

### DS-evoked responses in VP reflect response vigor and lever proximity

A large and redundant set of locomotor variables (Table 1) was computed for each approach to the lever following DS presentation. To determine which of these locomotor variables were reflected in the neural response to the DS, we first identified a subset of variables to be used as regressors in a GLM with neural firing as the response variable. To identify these variables, we applied PCA/FA of the locomotor variables. Using Kaiser’s criterion (eigenvalue > 1), we selected five factors for further examination (Fig. 2*A*). We chose a single representative variable that was strongly correlated with each of the first four factors as representative of that factor (Table 3). No variables were strongly correlated with the fifth factor, which had an eigenvalue very close to 1, and therefore we did not choose a representative variable for this factor. Each of the representative variables chosen had the highest “rep score” (the variable’s highest factor loading minus the sum of its loadings on all other factors) for its factor, an indication of how selectively the variable loads onto the factor. The chosen variables were maximum radial velocity with respect to the lever, movement duration, SD of angular velocity with respect to the operandum, and path efficiency.

**Table 3:** Factor loadings

	Factor 1	Factor 2	Factor 3	Factor 4	Factor 5		Rep score
**Maximum of radial velocity (with respect to operandum)**	**0.94**	0.25	0.16	0.01	−0.03		0.55
SD of radial velocity (with respect to operandum)	**0.92**	0.14	0.13	0.30	0.00		0.36
SD of speed	**0.88**	0.10	0.37	0.22	0.00		0.18
Maximum speed	**0.88**	0.24	0.39	−0.02	0.02		0.25
**Move duration**	0.09	**0.95**	0.06	−0.07	0.08		0.78
Path length	0.34	**0.82**	0.29	0.33	0.05		−0.18
Latency to maximum speed	0.04	**0.77**	0.09	−0.08	−0.02		0.73
Latency to maximum acceleration	0.04	**0.75**	0.08	−0.09	0.00		0.72
Maximum deviation from direct path	0.21	0.66	0.28	0.58	0.10		−0.50
**SD of angular velocity (with respect to operandum)**	0.39	0.12	**0.87**	0.17	0.00		0.19
Maximum of angular velocity (with respect to operandum)	0.45	0.23	**0.82**	−0.02	0.19		−0.02
Mean of angular velocity (with respect to operandum)	0.18	0.23	**0.74**	0.45	−0.38		0.26
**Turn efficiency**	0.01	0.04	0.04	−0.20	0.02		NA
Mean radial velocity (with respect to operandum)	0.54	0.20	0.14	**0.77**	0.20		−0.30
Mean speed	0.49	0.24	0.37	**0.75**	0.01		−0.36
**Path efficiency**	−0.03	−0.02	0.11	0.48	−0.02		0.43
**Movement onset latency**	−0.12	0.01	−0.04	−0.15	0.01		NA
**Net change in heading**	−0.01	−0.03	0.00	−0.01	0.01		NA

PCA followed by FA was used as the basis to select a subset of variables for subsequent use as regressors in GLMs to detect correlation with neuronal activity. Variables used as regressors are shown in bold. We selected a representative variable for each factor based on high loading onto its representative factor and low co-loading onto other factors (which together are represented by the rep score, the variable’s highest factor loading minus the sum of its loadings onto all other factors). These representative variables were: factor 1, maximum of radial velocity with respect to operandum; factor 2, move duration; factor 3: SD of angular velocity with respect to operandum; factor 4, path efficiency. Additionally, we selected three “independent” variables that did not load strongly onto any one factor (turn efficiency, net heading change, and movement onset latency). NA, not applicable.

In addition, we selected three variables that did not load strongly onto any factor (net change in heading, turn efficiency, and latency to movement onset; Table 3) as well as three non-motor variables that described the subject’s behavioral state at cue onset (distance from the operant lever, time elapsed since last cue, and time since last reward). In total, 10 variables were subsequently used as regressors to fit each neuron’s DS-evoked response in a GLM. Because we chose motor variables with the highest rep scores, multicollinearity between variable pairs was minimal, with no variable pair exhibiting a Pearson coefficient higher than 0.56 (Fig. 2*B*).

For each neuron, we fit a GLM to DS-evoked firing (50–500 ms after cue) using the 10 locomotor and behavioral state variables described above as regressors. In order to compare the resulting 10 regression estimates (β values) obtained for each neuron across neurons and across different regressors, we calculated the IDR Firing Difference for each regressor (estimated percentage difference in firing from the 10th to 90th percentile value of the regressor; see Materials and Methods). Figure 3 shows the means of IDR Firing Differences across the 98 DS-excited neurons that met the criteria for analysis (see Materials and Methods; also see Table 4 for detailed statistical results). We modelled the overall response (40–400 ms after cue onset) as well as the early phase of the response (40–180 ms) and the late phase (180–400 ms, capturing the second, minor firing peak).

**Table 4:** Statistical summary table

Comparison	Figure	Test	Statistic	*p* value	Multiple comparison procedure
DS-evoked firing vs NS-evoked firing	1*F*	Wilcoxon	*V* = 18013	*p* < 0.0001	None
DS-evoked firing: behavioral response vs no response	n/a	Wilcoxon	*V* = 11007	*p* < 0.0001	None
Large model, overall firing window tests for IDR firing difference from 0					
Maximum radial velocity	3*A*,*B*	*t*	2.53	*p* = 0.13	Holm
Move duration	3*A*	*t*	1.33	*p* = 0.19	Holm
SD of angular velocity	3*A*	*t*	0.43	*p* = 0.67	Holm
Path efficiency	3*A*,*B*	*t*	2.61	*p* = 0.10	Holm
Net Δ in heading	3*A*	*t*	1.67	*p* = 0.10	Holm
Turn efficiency	3*A*	*t*	0.50	*p* = 0.62	Holm
Latency to move	3*A*	*t*	−1.40	*p* = 0.17	Holm
Distance from lever	3*A*,*B*	*t*	−1.37	*p* = 0.17	Holm
Time since reward	3*A*	*t*	−0.96	*p* = 0.34	Holm
Time since cue	3*A*	*t*	1.60	*p* = 0.11	Holm
Large model, early firing window tests for IDR firing difference from 0					
Maximum radial velocity	3*C*,*D*	*t*	1.75	*p* = 0.084	Holm
Move duration	3*C*	*t*	1.95	*p* = 0.054	Holm
SD of angular velocity	3*C*	*t*	0.92	*p* = 0.36	Holm
Path efficiency	3*C*,*D*	*t*	3.48	*p* = 0.00075	Holm
Net Δ in heading	3*C*	*t*	1.56	*p* = 0.12	Holm
Turn efficiency	3*C*	*t*	−0.76	*p* = 0.45	Holm
Latency to move	3*C*	*t*	−1.52	*p* = 0.13	Holm
Distance from lever	3*C*,*D*	*t*	0.65	*p* = 0.52	Holm
Time since reward	3*C*	*t*	−0.35	*p* = 0.73	Holm
Time since cue	3*C*	*t*	0.71	*p* = 0.48	Holm
Large model, late firing window tests for IDR firing difference from 0					
Maximum radial velocity	3*E*,*F*	*t*	3.48	*p* = 0.00075	Holm
Move duration	3*E*	*t*	1.00	*p* = 0.32	Holm
SD of angular velocity	3*E*	*t*	0.64	*p* = 0.52	Holm
Path efficiency	3*E*,*F*	*t*	0.75	*p* = 0.45	Holm
Net Δ in heading	3*E*	*t*	1.38	*p* = 0.17	Holm
Turn efficiency	3*E*	*t*	2.74	*p* = 0.0074	Holm
Latency to move	3*E*	*t*	0.27	*p* = 0.79	Holm
Distance from lever	3*E*,*F*	*t*	−3.66	*p* = 0.00042	Holm
Time since reward	3*A*	*t*	0.62	*p* = 0.54	Holm
Time since cue	3*A*	*t*	1.27	*p* = 0.21	Holm
Comparison of IDR firing response across rats					
Path efficiency, early excitation window	5*A*	ANOVA	*F* = 0.8727	*p* = 0.458	
Maximum radial velocity, late excitation window	5*B*	ANOVA	*F* = 0.32	*p* = 0.812	
Lever distance, late excitation window	5*C*	ANOVA	*F* = 6.84	*p* = 0.0003	
Focused model, overall firing window tests for IDR firing difference from 0					
Maximum radial velocity	6*A*,*B*	*t*	2.18	*p* = 0.032	Holm
Path efficiency	6*A*,*B*	*t*	2.22	*p* = 0.029	Holm
Distance from lever	6*A*,*B*	*t*	−1.64	*p* = 0.10	Holm
Focused model, early firing window tests for IDR firing difference from 0					
Maximum radial velocity	6*C*,*D*	*t*	1.24	*p* = 0.22	Holm
Path efficiency	6*C*,*D*	*t*	3.26	*p* = 0.0015	Holm
Distance from lever	6*C*,*D*	*t*	0.46	*p* = 0.65	Holm
Focused model, late firing window tests for IDR firing difference from 0					
Maximum radial velocity	6*E*,*F*	*t*	2.42	*p* = 0.017	Holm
Path efficiency	6*E*,*F*	*t*	0.69	*p* = 0.49	Holm
Distance from lever	6*E*,*F*	*t*	−4.33	*p* = 0.000037	Holm
Regression of Q2 vs Q1 early firing Z scores for no infusion					
Intercept	8*F*	*t*	0.62	*p* = 0.54	None
Slope	8*F*	*t*	9.89	*p* = 3.59 × 10^−13^	None
Slope vs 1	8*F*	*t*	−1.03	*p* = 0.31	None
Regression of Q2 vs Q1 early firing Z scores for bilateral vehicle					
Intercept	8*G*	*t*	0.36	*p* = 0.72	None
Slope	8*G*	*t*	4.50	*p* = 0.00022	None
Slope vs 1	8*G*	*t*	−0.07	*p* = 0.945	None
Regression of Q2 vs Q1 early firing Z scores for contralateral SCH-23390					
Intercept	8*H*	*t*	0.108	*p* = 0.915	None
Slope	8*H*	*t*	5.92	*p* = 4.87e-6	None
Slope vs 1	8*H*	*t*	0.35	*p* = 0.73	None
Regression of Q2 vs Q1 early firing Z scores for ipsilateral SCH-23390					
Intercept	8*I*	*t*	*t* = 1.23	*p* = 0.23	None
Slope	8*I*	*t*	*t* = 5.81	*p* = 2.68e-6	None
Slope vs 1	8*I*	*t*	−1.12	*p* = 0.27	None
Regression of Q2 vs Q1 early firing Z scores for bilateral SCH-23390					
Intercept	8*J*	*t*	−0.04	*p* = 0.97	None
Slope	8*J*	*t*	3.79	*p* = 0.0016	None
Slope vs 1	8*J*	*t*	−8.37	*p* = 3.09e-7	None
Regression of Q2 vs Q1 late firing Z scores for no infusion					
Intercept	8*K*	*t*	0.28	*p* = 0.78	None
Slope	8*K*	*t*	10.71	*p* = 2.53e-14	None
Slope vs 1	8*K*	*t*	−1.25	*p* = 0.22	None
Regression of Q2 vs Q1 late firing Z scores for bilateral vehicle					
Intercept	8*L*	*t*	0.79	*p* = 0.44	None
Slope	8*L*	*t*	8.44	*p* = 5.08e-8	None
Slope vs 1	8*L*	*t*	−0.18	*p* = 0.857	None
Regression of Q2 vs Q1 late firing Z scores for contralateral SCH-23390					
Intercept	8*M*	*t*	0.51	*p* = 0.61	None
Slope	8*M*	*t*	6.41	*p* = 1.53e-6	None
Slope vs 1	8*M*	*t*	−2.36	*p* = 0.027	None
Regression of Q2 vs Q1 late firing Z scores for ipsilateral SCH-23390					
Intercept	8*N*	*t*	0.80	*p* = 0.43	None
Slope	8*N*	*t*	8.16	*p* = 5.37e-9	None
Slope vs 1	8*N*	*t*	−1.10	*p* = 0.28	None
Regression of Q2 vs Q1 late firing Z scores for bilateral SCH-23390					
Intercept	8*O*	*t*	−1.60	*p* = 0.13	None
Slope	8*O*	*t*	5.72	*p* = 3.17e-5	None
Slope vs 1	8*O*	*t*	−8.61	*p* = 2.13e-7	None

List of statistical comparisons and results.

To identify which regressors were correlated with the neural population response, we asked whether the mean of each regressor’s IDR Firing Difference was different from 0 (one-sample *t* test using Holm’s modified Bonferroni correction). In the overall response window (40–400 ms), no regressor’s IDR Firing Difference was found to be significantly different from 0 (Fig. 3*A*,*B*). However, in the early response window (40–180 ms), the IDR Firing Difference for path efficiency was significantly more positive than 0 (Fig. 3*C*,*D*). In the late response window (180–400 ms) the IDR Firing Difference for maximum radial velocity toward the lever was significantly more positive than 0, and the IDR Firing Difference for distance from the lever at the time of cue-onset was significantly more negative than 0 (Fig. 3*E*,*F*). Representative rasters and histograms for DS-evoked firing of two neurons illustrate the relationship between firing and distance from the lever at cue onset (Fig. 4). Histograms showing the distributions of β values for the three significant variables are shown in Figure 3*B*,*D*,*F*; gray shaded bars indicate neurons for which the regressor’s contribution to the neuron’s model was significant.

To test whether these significant effects were potentially the result of oversampling neurons from individual rats that showed aberrantly strong representations of one or more variables, we used one-way ANOVAs to compare the mean IDR Firing Difference across rats. In the case of path efficiency in the early excitation window and maximum radial velocity in the late window, there were no significant differences across animals and the mean IDR Firing Difference was positive for each animal (Fig. 5*A*,*B*), indicating that the significantly positive IDR Firing Differences in the population analysis (Fig. 6) were consistently observed across subjects. In the case of distance from the lever, the cross-animal ANOVA comparison was significant. All but one subject showed a strongly negative mean IDR Firing Difference, and even in the remaining subject, the mean IDR Firing Difference was close to 0 and many values were negative (Fig. 5*C*). Therefore, the negative mean IDR Firing Difference for lever distance observed in the population analysis (Fig. 6) is clearly not because of overrepresentation of neuronal data from a single animal.

To verify these results, we applied the same GLM approach restricted to only the three regressors that were significant in any response window in the overall GLM (Fig. 6). We found that in the overall window, maximum radial velocity and path efficiency were positively correlated with cue-evoked excitations, while lever distance was significantly negatively correlated. In the early response window, radial velocity and path efficiency were significantly positively correlated, and in the late response window, radial velocity was positively correlated, while distance from the lever at cue-onset was negatively correlated with cue-evoked excitation. Although a greater number of comparisons crossed the significance threshold in the focused model, the signs of the effects were similar across the larger and focused models, and in no case did the focused model fail to replicate a significant result observed in the larger model. Therefore, the results of the focused GLM are consistent with the results from the larger GLM.

### Unilateral infusion of SCH-23390 in the NAc does not affect VP response to the DS or locomotor encoding

**Figure 7.**
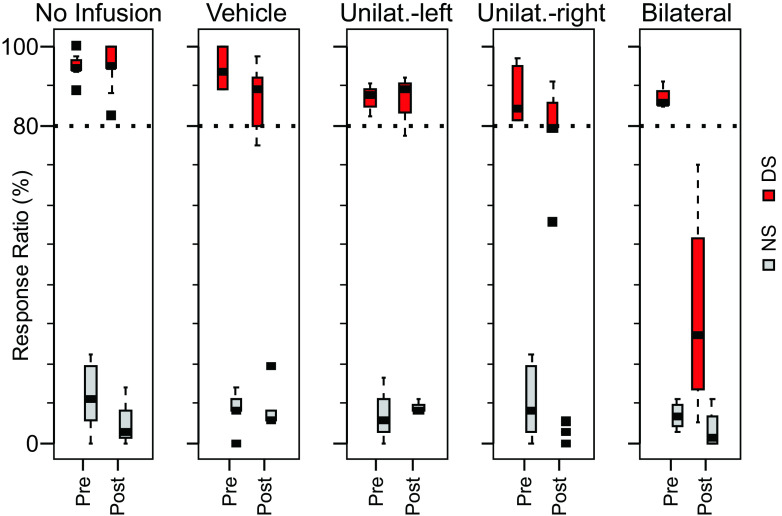
Bilateral but not unilateral infusion of the D1 antagonist SCH-23390 into the NAc severely impairs behavioral performance. Box plots show the mean (horizontal line), interquartile range (box), extremes (whisker), and outliers (points) of the response ratio for the indicated cue (DS or NS) preinfusion and postinfusion of the indicated drug (for detailed description of box-and-whisker plots, see legend to Fig. 5). Unilat., unilateral.

**Figure 8.**
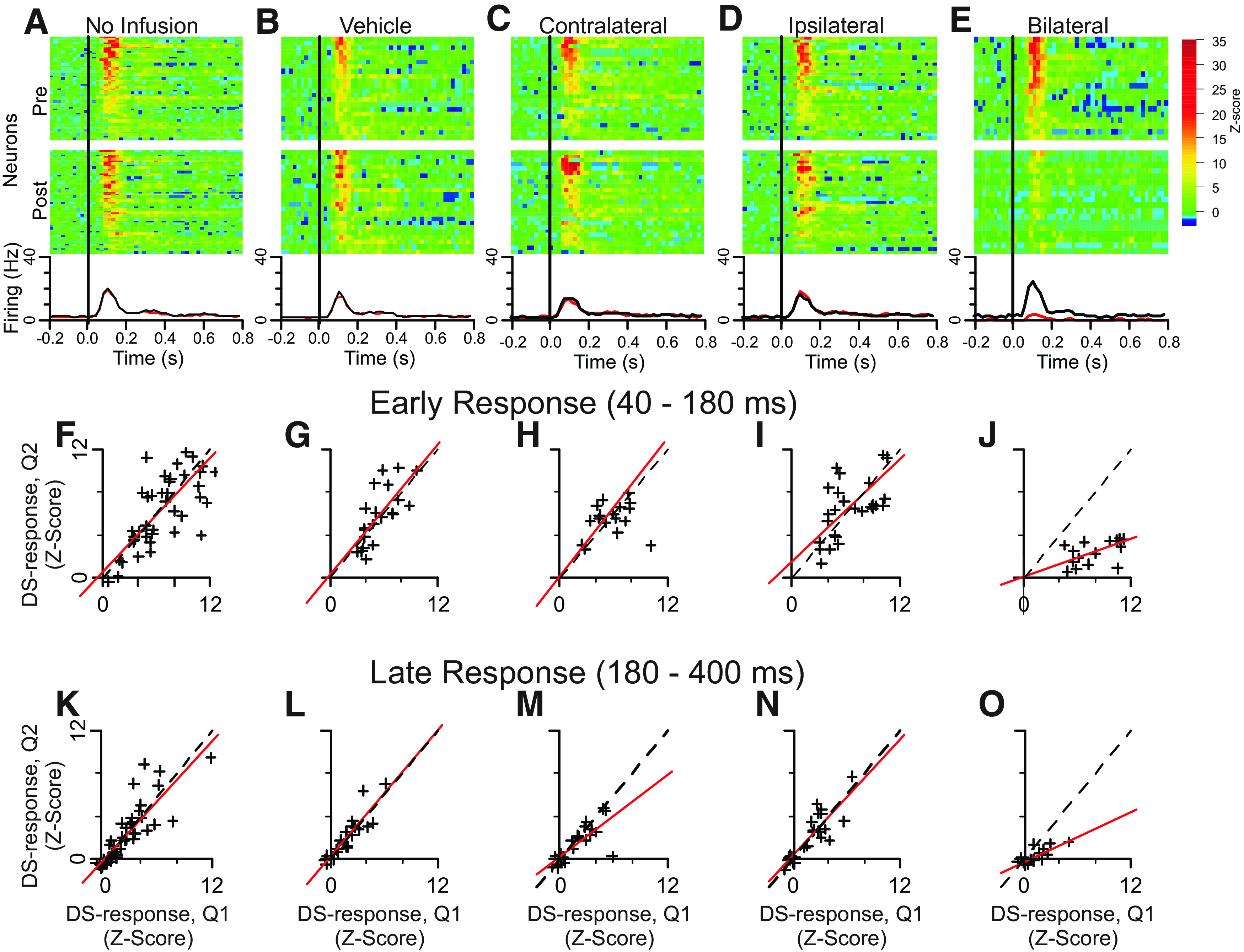
The D1 antagonist SCH-23390 infused into the NAc does not significantly alter VP neuronal responses to DS onset, except when infused bilaterally. ***A–E***, Heat maps depicting firing responses of neurons that were significantly activated by DS onset. Data from the same neurons (in the same order) are shown before (pre, upper) and after (post, lower) infusion. ***F–J***, Correlation of mean Z-score of response in the 40- to 180-ms window following DS-onset before and after infusion. ***K–O***, Same as ***F–J*** but for the response window of 180–400 ms following cue-onset.

To assess the role of dopamine-dependent NAc input on cue-evoked VP firing, we infused SCH-23390, a D1-receptor antagonist, into the NAc of all four rats via an infusion cannula during the task after a 45-min preinjection control period. After a control period of 45 min (25% of the total session duration), bilateral SCH-23390 (*N* = 4 sessions; *N* = 38 DS-excited neurons), unilateral SCH-23390 (*N* = 3 left, 4 right hemisphere injection sessions; *N* = 31 DS-excited neurons recorded ipsilateral to the injection, 26 contralateral), or bilateral vehicle (*N* = 4 sessions; *N* = 19 DS-excited neurons) infusions were made; in addition, we considered sessions in which no injections were made (*N* = 14 sessions; *N* = 50 DS-excited neurons). The DS response ratio remained above the criterion level of 0.80 in all groups except the bilaterally SCH-23390 infused group, for which the DS response ratio was severely reduced (Fig. 7). The effects of these infusions on cue-evoked excitations were visualized with heat maps of the mean firing Z-score (comparing firing to a 1-s precue control period). Heat maps were constructed separately for preinjection and postinjection windows (45 min, ∼25 trials each; Fig. 8*A–E*). To quantify these effects, we first compared firing in a postcue window of 40–180 ms after cue onset in the 45-min epoch after drug infusion versus the 45-min epoch before infusion by regressing, across neurons, firing in these two epochs. Firing was calculated as the Z-score of postcue firing rate relative to the 1-s precue baseline and the Z-scores obtained in the 45 min after the infusion (Q2) were regressed against those obtained in the 45-min preinfusion baseline (Q1; Fig. 8*F–J*). For all injection conditions, the intercepts were not significantly different from 0 (*p*_no-infusion_ = 0.95, *p*_vehicle_ = 0.20, *p*_contra_ = 0.08, *p*_ipsi_ = 0.26, *p*_bilateral_ = 0.55). The coefficients (β) were significant in all groups (*p*_no-infusion_ = 0 < 0.001, *p*_vehicle_ < 0.001, *p*_contra_ < 0.001, *p*_ipsi_ < 0.001, *p*_bilateral_ < 0.01); however, only in the case of bilateral infusion was the coefficient significantly different from 1 (β ± SE: no-infusion, 0.88 ± 0.10; vehicle, 1.00 ± 0.22; contralateral, 1.06 ± 0.25; ipsilateral, 0.79 ± 0.18; bilateral, 0.30 ± 0.09).

Likewise, there was no evidence for an effect of unilateral SCH-23390 injection on the late phase response (180–400 ms after DS-onset; Fig. 8*K–O*). Using the same method of analysis described above we found that again, only bilateral infusion had any effect on cue-evoked excitations. Intercepts for regression models were not significantly different from 0 (*p*_no-infusion_ = 0.51, *p*_vehicle_ = 0.74, *p*_contra_ = 0.33, *p*_ipsi_ = 0.47, *p*_bilateral_ = 0.93). The coefficients (β) were significant in all groups except the vehicle control group (*p*_no-infusion_ = 0 < 0.001, *p*_vehicle_ = 0.11, *p*_contra_ < 0.001, *p*_ipsi_ < 0.001, *p*_bilateral_ < 0.05); however, as we found for the early phase response, only in the case of bilateral infusion was the coefficient significantly different from 1 (β ± SE: no-infusion, 0.93 ± 0.12; vehicle, 0.80 ± 0.45; contralateral, 0.78 ± 0.14; ipsilateral, 0.86 ± 0.16; bilateral, 0.34 ± 0.10).

### Probe localization

**Figure 9.**
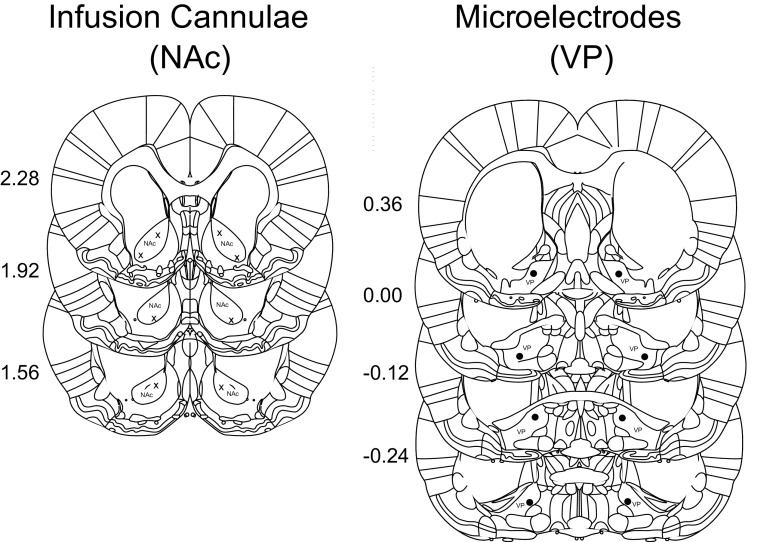
Recording electrode (X) and infusion cannula sites (solid circles) were localized following completion of experimental session from 20-μm sections. Coordinates were determined by comparison with nearby landmark features (Paxinos and Watson, 1998). Numbers indicate anteroposterior distance of the coronal section from bregma (mm).

Histologic determination of probe locations showed that electrodes were located in the VP and cannulae in the NAc core (Figure 9).

## Discussion

Consistent with previous observations (Richard et al., 2016, 2018), we found that nearly half of VP neurons were significantly excited by auditory cues predicting sucrose reward (DS), and that these excitations were smaller in response to cues that did not predict reward (NS). Animals almost always responded to DS presentation by approaching and then pressing the active lever, causing reward to be delivered. Importantly, the interval between DS presentations was both long (mean of ∼60 s) and variable. Because the subjects could not predict when the next DS would be presented, they moved about the operant chamber such that their location at cue onset varied (data not shown; see Nicola, 2010). Therefore, reaching the lever after cue onset required the animal to determine and execute a different movement path on each trial. VP neurons, which are required for the cued approach response in a similar behavioral task (Richard et al., 2016, 2018), could contribute a component of the movement path computation. Alternatively, VP neurons could serve some other function, such as activating or invigorating approach behavior in response to information that reward is likely to be available. Finally, if cue-evoked excitations begin after movement onset, the firing could report motor or spatial information resulting from movement.

Consistent with the former two hypotheses, we found that the cue-evoked excitations of VP neurons reliably preceded approach movement initiation, consistently beginning ∼50 ms after cue onset whereas locomotion typically began >400 ms after cue onset (Fig. 1*B–D*). Excitations consisted of a large early peak that ended ∼180 ms after cue onset, followed by a smaller but longer tail that extended at least 400 ms past cue onset (Fig. 1*C*). Excitations were sharply aligned to cue presentation (and not movement onset) even for the trials with the longest latency to initiate movement (Fig. 1*D*). Therefore, cue-evoked excitations are temporally situated such that they could influence cued approach movement, rather than reporting information related to ongoing movements or their consequences.

We assessed the nature of this potential contribution by determining how firing was related to the characteristics of the lever approach movement. To do this, we first computed multiple parameters for each cued lever approach movement. These parameters included those related to speed, duration, efficiency, and turn direction (Table 1). Next, we assessed the underlying correlation structure among these parameters using PCA/FA, and identified four parameters that uniquely represent individual principal components while being only weakly influenced by the other components (maximum of radial velocity with respect to the lever; movement duration; SD of angular velocity with respect to the lever; and path efficiency). Three additional movement parameters (net change in heading, turn efficiency and locomotion onset latency) were not correlated with any of the four components. Thus, we identified seven representative movement parameters that together account for most of the variance in the movement parameter space; none of these parameters were strongly correlated with each other (Fig. 2*B*). Some of these variables represent characteristics of the structure of the movement (change in heading), others represent characteristics of movement vigor (radial and angular velocity), and others are “hybrid” variables that could be influenced by both (path and turn efficiency, movement duration). We included three additional independent variables for further analysis, representing the animal’s state at cue onset (distance from lever, time since last reward, time since last cue presentation).

Next, we used these 10 parameters as independent variables for three GLMs per neuron, each with the firing rate in a different window after cue onset as the dependent variable. To assess whether individual variables were consistently encoded by firing across neurons, we used the variable’s β values to determine each neuron’s percent change in firing across the IDR of the variable (the IDR Firing Difference). In the firing analysis window 40–400 ms after cue onset (comprising both the primary excitation peak and the first and largest part of the tail), no parameters exhibited mean IDR Firing Differences that were significantly different from 0 (Fig. 3*A*). Therefore, although the β values for some individual neurons were significant for some variables (Fig. 3*B*), there were no variables with a consistent relationship to firing in the 40- to 400-ms window. When we limited the analysis window to the early excitation peak (early firing response, 40–180 ms after cue onset), we found that path efficiency was strongly related to the firing response. The mean IDR Firing Difference was significantly positive, meaning that the early firing peak tended to be greater preceding more efficient movements (Fig. 3*C*,*D*). During the secondary peak and tail of the excitation (late firing response, 180–400 ms), firing was consistently greater when the maximum radial velocity was higher, and consistently smaller when distance from the lever at cue onset was greater (Fig. 3*E*,*F*).

These results were confirmed using a focused GLM that included only maximum radial velocity, path efficiency, and distance from the lever at cue onset (Fig. 6). The focused GLM results recapitulated all significant effects from the larger GLM. In addition, all three variables exhibited significant effects in the overall time window (40–400 ms), and maximum radial velocity was positively related in both the early and late windows. That these effects were significant in the focused but not larger model suggests that the effects are partly accounted for by other variables in the larger model, although no other variables were consistently encoded by the population as a whole. We conclude that VP neurons most consistently showed higher peak cue-evoked firing when the efficiency of the subsequent movement was higher, as well as greater cue-evoked firing in the tail of the response when the animal’s location at cue onset was closer to the lever and when the velocity of the lever-directed movement was faster. These results are consistent with the hypothesis that VP neurons’ cue-evoked firing promotes more vigorous and direct approach movements. In addition, the tail of the excitation reflects the animal’s starting proximity to the movement target, similar to the cue-evoked firing of NAc neurons (McGinty et al., 2013; Morrison et al., 2017).

Some of the variables used in the GLM analysis were correlated with each other to a degree (e.g., lever distance with movement duration, path efficiency, radial velocity and angular velocity; movement duration with radial velocity and angular velocity; radial velocity with angular velocity; Fig. 2*B*), similar to previous observations in rats performing a similar task (McGinty et al., 2013; Morrison et al., 2017; Richard et al., 2018). Superficially, these correlations suggest that VP neurons could encode a single aspect of movement (e.g., radial velocity) and exhibit correlations with other parameters (path efficiency, distance from lever) by virtue of the relationships among the independent variables. However, GLM coefficients represent the strength of the relationship between the movement variable and firing rate after accounting for the influence of other variables. Moreover, although lever distance was positively related to radial velocity and path efficiency, the relationship between firing and lever distance (negative) was in the opposite direction to the relationship between firing and radial velocity and path efficiency (both positive). Finally, whereas radial velocity was related to firing throughout the postcue period, path efficiency was related to firing only during the peak, and lever distance was related to firing only during the tail. Therefore, the influence of each of these three variables on VP neurons is independent of the influence of the others.

Consistent with our observations, previous studies have observed strong cue-evoked excitations in VP neurons during similar tasks. As in our study, these excitations reflected the proximity of the animal to the movement target at cue onset, with greater firing when the animal was closer to the target (Richard et al., 2016, 2018). In addition, greater cue-evoked firing was observed when subjects reached the target sooner, an effect that appeared to be explained by the fact that shorter latencies occur when the animal is closer to the target. However, our GLM analysis allowed us to separate the contributions of specific motor vigor parameters to firing. The results indicate that greater firing predicts greater speed and efficiency of the approach to the operandum, and that these effects are independent of any effect of proximity on firing. Thus, vigor encoding in VP neurons is not simply a function of proximity encoding but an additional contributor to firing.

Intriguingly, the latency of the animal to enter a reward receptacle in response to a Pavlovian cue predicting reward delivery 8 s after cue onset was not reflected in the cue-evoked firing of VP neurons (Richard et al., 2018), although VP neurons were excited by such cues. Although this result suggests that the vigor parameters identified here would also not be encoded by VP neurons during Pavlovian tasks, a key difference between typical Pavlovian and DS (instrumental) tasks is that the cue indicates delayed reward delivery in the Pavlovian case, but immediate reward delivery contingent on a response in the instrumental case. More vigorous responding therefore benefits the animal in the instrumental but not Pavlovian tasks. Therefore, a key role for VP neurons may be to invigorate approach behavior in response to information that reward is imminent. Notably, this could, in theory, occur even in Pavlovian situations if the Pavlovian conditioned stimulus predicts an immediate rather than delayed unconditioned stimulus.

The movement target proximity and predictive vigor encoding described here is similar to that previously observed in the NAc, where greater cue-evoked firing predicted shorter latency to initiate movement and faster approach speed, and cue-evoked firing increased as the starting distance to the operandum decreased (McGinty et al., 2013; Morrison and Nicola, 2014; Morrison et al., 2017). One important difference is that we found little evidence of encoding of latency to initiate approach movement in VP neurons. These results suggest that VP neurons’ firing activity does not influence movement initiation, but could influence the speed of approach movement. Consistent with these observations, VP inactivation increases the latency to perform operant responses to cues (Richard et al., 2018). Our results suggest that this effect is because of decreased speed of approach movement rather than a greater latency to initiate movement.

Because the NAc projects strongly to the VP, the similarity in their neurons’ speed and proximity encoding raises the possibility that the encoding of these parameters by VP neurons is partly because of the influence of NAc neurons. Arguing against this, cue-evoked excitations in the VP begin before those that have been observed in the NAc (Richard et al., 2016). Confirming this observation, we found the excitation latency after cue onset is <60 ms in the vast majority of VP cue-excited neurons, whereas it >70 ms in half of NAc cue-excited neurons (and the latency to inhibition is even longer in NAc cue-inhibited neurons) in animals performing a nearly identical behavioral task (Morrison et al., 2017). However, NAc and VP excitations overlap, leaving open the possibility that NAc cue-evoked excitations influence ongoing VP activity (perhaps via a polysynaptic circuit including an inhibitory synapse, as NAc projection neurons are GABAergic and their excitations would be expected to result in direct inhibition of VP neurons). To test this possibility, we injected the dopamine D1 receptor antagonist SCH-23390 into the NAc, a manipulation that uniformly blunts the cue-evoked excitations of NAc neurons (du Hoffmann and Nicola, 2014) when performed either unilaterally or bilaterally. Unilateral injections had minimal behavioral effects, but bilateral injections severely impaired DS responding, as reported previously (du Hoffmann and Nicola, 2014). VP cue-evoked excitations were also not affected by unilateral NAc injections, but were severely reduced in amplitude after bilateral injections (Fig. 8). These results indicate that it is unlikely that NAc cue-evoked excitations influence those in the VP. However, it remains formally possible that a strong projection from the contralateral NAc contributes to VP cue-evoked firing only in the absence of an intact ipsilateral projection.

The D1 antagonist experiment leaves open two further possibilities. First, NAc cue-evoked excitations could contribute directly to cue-evoked inhibitions of VP neurons. Small populations of such inhibitions have been reported previously (Richard et al., 2016, 2018), but very few were observed here, perhaps because of differences in electrode location. Second, some NAc neurons exhibit cue-evoked inhibitions (Nicola et al., 2004; Morrison et al., 2017), which could, in theory, contribute directly to VP neurons’ excitations. In contrast with NAc neurons’ cue-evoked excitations, D1 antagonist treatment of the NAc has little if any effect on cue-evoked inhibitions in the NAc (du Hoffmann and Nicola, 2014). Therefore, the absence of ipsilateral NAc D1 antagonist treatment effects on VP neurons’ cue-evoked excitations does not rule out the possibility that inhibitions in the NAc contribute to excitations in the VP. On the other hand, cue-evoked inhibitions in the NAc begin at even longer latency after cue onset than cue-evoked excitations (90 ms; Morrison et al., 2017), well after VP excitations have begun (typically <60 ms).

Together with previous findings, our results suggest that a serialized model of ventral basal ganglia function, where each nucleus performs a function and activates or inhibits “downstream” nuclei in turn, is not accurate, at least in the case of operant DS tasks. Instead, we favor a model in which the NAc and VP receive simultaneous activation by inputs conveying similar but not identical information, and these structures work in parallel to set the vigor of the upcoming approach response. Future studies should examine which upstream structures convey cue-evoked activity to the basal ganglia; for example, the hippocampus, medial prefrontal cortex, and sensory thalamic areas are obvious candidates. Moreover, because the strong and direct NAc-VP connection appears to play little if any role in cued approach behavior, future studies must also establish the behavioral function of this connection.

## Synthesis

Reviewing Editor: Alexxai Kravitz, Washington University in St. Louis Decisions are customarily a result of the Reviewing Editor and the peer reviewers coming together and discussing their recommendations until a consensus is reached. When revisions are invited, a fact-based synthesis statement explaining their decision and outlining what is needed to prepare a revision will be listed below. The following reviewer(s) agreed to reveal their identity: Jocelyn Richard.

Critical comments to addess:

1) The authors should specify and clearly describe important methodological details in the results. These include sex of animals, number of animals recorded in each experiment, and number of units recorded in each experiment in the paper.

2) The histology figure would be enhanced by inclusion of at least one photograph so a reader can assess the spatial extend of the recording arrays in the VP.

3) The authors should describe some information on the neurons that were excluded from this analysis of DS responsive units. Namely, did units that did not change firing rate to the DS change firing at the onset of locomotion? In addition it would be helpful to present a direct graphical representation of the association between firing and motor parameters.

4) Related to 2, were motor parameters, such as path efficiency and radial velocity, affected by D1 manipulations?

5) The authors are encouraged to make data and code available after publication. This is required to comply with eNeuro policy, as is including a heading in the methods describing “Code Availability"

***********************

Reviewer 1

***********************

In this manuscript the authors examine how cue-evoked ventral pallidum (VP) activity relates to a broad range of movement parameters, and whether VP cue responses are impacted by nucleus accumbens (NAc) D1 dopamine receptor (D1R) antagonism, which has been shown disrupted NAc cue responses. They find that cue-evoked excitations in VP neurons encode vigor parameters (speed and efficiency of movement) and proximity to movement target, but not other movement parameters, such as movement onset latency, which have been shown to be encoded by the NAc. Additionally, while bilateral NAc D1R antagonism disrupts both behavioral responding and VP cue excitations, ipsilateral NAc D1R antagonism does not significantly impact VP cue responses, suggesting that NAc activity changes are not directly responsible for cue responses in the VP. Overall, this is a solid and scholarly manuscript, with robust GLM analyses and appropriate controls. I only have a few minor comments.

1. Overall, the statistical analyses, especially the GLM, are comprehensive and well described. My one concern is that some tests that rely on an assumption of independence are run on the single unit data (e.g. assessing whether the modeled differences in firing rate across the population are different from 0 with a ttest), when the these data are not fully independent. It would strengthen the conclusions of the manuscript to include analyses that account for the random effect of subject when assessing whether these values are different from 0 across the population.

2. It would be helpful to know how many neurons were recorded from each subject, and how many neurons are included in the analysis of the dopamine antagonist effects. Additionally, how many subjects were tested with ipsilateral injections? The manuscript says that n=3 for left, and n=4 for right, but it wasn’t clear how that corresponding to ipsilateral versus contralateral.

3. How was the 180ms time point selected for the end of the early window?

4. The authors should specify that they are using male rats in the abstract.

5. I would strongly encourage the authors to make their data and code available upon publication.

***********************

Reviewer 2

***********************

Ventral pallidum neurons fire during different aspects of motivated behavior. The authors investigated whether pallidal firing following a reward-related cue was influenced by locomotion as well as whether the firing following the cue predicted motor parameters such as velocity. A role for D1R in accumbens was tested in the firing patterns of pallidal neurons.

The manuscript is fairly limited in scope, only examining a subset of recorded neurons that increased firing following the DS. These neurons did not strongly increase firing by locomotion initiation. Were there neurons not increasing in firing rate to the DS that changed firing at locomotion onset?

The statistical model showed associations between cue-evoked firing and radial velocity, path efficiency, distance from lever (180-400ms). While the presentation of this data is adequate, it would be helpful to show correlations of firing rate changes with the motor parameters or at least a more direct graphical representation of firing with the motor parameters.

I encourage the authors to think about removing the phrase “encode” from the manuscript. While the neurons studied have an association between the behaviors studied and firing rate, many other factors could influence firing rate that were not tested (breathing changes, heart rate changes, etc). We simply don’t know what these neurons “encode", only what behaviors are associated with increases or decreases in firing rate from baseline. The use of “encoding” without testing dilutes from the results and leads our field down a path of confusing interpretations.

Were motor parameters, such as path efficiency and radial velocity, affected by D1 manipulations?

Histology figure is inadequate for replication. Were there any regional variations?

minor - fig 1a define acronym

- fig 1f a legend key defining the shape of each point would be useful

- fig 6 no legend for color firing plot
